# Deep Learning Based Egg Fertility Detection

**DOI:** 10.3390/vetsci9100574

**Published:** 2022-10-17

**Authors:** Kerim Kürşat Çevik, Hasan Erdinç Koçer, Mustafa Boğa

**Affiliations:** 1 Faculty of Applied Sciences, Akdeniz University, Antalya 07070, Turkey; 2 Faculty of Technology, Selçuk University, Konya 42130, Turkey; 3 Bor Vocational School, Niğde Ömer Halisdemir University, Niğde 51700, Turkey

**Keywords:** deep learning, egg fertility, Mask R-CNN, incubator images

## Abstract

**Simple Summary:**

This study employs a Mask R-CNN technique along with the transfer learning model to accurately detect fertile and infertile eggs. It is a novel study that uses a single DL model to carry out detection, classification and segmentation of fertile and infertile eggs based on incubator images.

**Abstract:**

This study investigates the implementation of deep learning (DL) approaches to the fertile egg-recognition problem, based on incubator images. In this study, we aimed to classify chicken eggs according to both segmentation and fertility status with a Mask R-CNN-based approach. In this manner, images can be handled by a single DL model to successfully perform detection, classification and segmentation of fertile and infertile eggs. Two different test processes were used in this study. In the first test application, a data set containing five fertile eggs was used. In the second, testing was carried out on the data set containing 18 fertile eggs. For evaluating this study, we used AP, one of the most important metrics for evaluating object detection and segmentation models in computer vision. When the results obtained were examined, the optimum threshold value (IoU) value was determined as 0.7. According to the IoU of 0.7, it was observed that all fertile eggs in the incubator were determined correctly on the third day of both test periods. Considering the methods used and the ease of the designed system, it can be said that a very successful system has been designed according to the studies in the literature. In order to increase the segmentation performance, it is necessary to carry out an experimental study to improve the camera and lighting setup prepared for taking the images.

## 1. Introduction

Poultry production is increasing every day in terms of meeting animal protein and other nutritional needs, which are important for humans around the world. In addition to the importance of poultry products in terms of the growth, development and healthy life of people, it is more economical, and it has a lower cholesterol content than red meat. This situation causes it to be preferred over poultry meat. In addition, it is an important advantage that more animal products are obtained from the unit area, and the unit cost is more economical. This situation increases chicken production in our country and worldwide, and the use of sensitive livestock and information communication technologies in this regard increases every day. Increasing animal production is important in terms of improving incubation conditions, detecting fertile eggs early, making production more economical and providing more egg output by using the available opportunities.

The studies carried out to improve this situation and the use of technology are increasing every day. Recently, it has been stated that early hatching power can be predicted by improving hatchability with the use of artificial intelligence, machine learning and different technological opportunities [1,2,3,4]. With the use of such applications, electricity and space savings will be achieved by preventing the unnecessary waiting for infertile eggs in the incubator with early diagnosis in the incubator. This situation provides great economic returns to businesses.

Adegbenjo et al. [5] reported that a fast and online prediction technology is needed, and different methods can be used to help the early identification of chicken egg output power. For this reason, in their study, they examined current approaches such as ultrasound and dielectric measurement, thermal imaging, machine vision, spectroscopy and hyperspectral imaging. They also reported that care should be taken to obtain quality data with more sample sizes in the relevant categories and to use appropriate analysis/modeling and evaluation techniques. Physical parameters of the egg (such as the size of the egg, shape index, shape and thickness of the shell) affect the output power. For this purpose, they used Minitab to determine the egg output power by using image processing and fuzzy logic for the physical properties of chicken eggs. The results can be used to determine the effect of the output power of the physical properties of the egg, to determine the ratio of all image processing, fuzzy logic and K-NN output power [1]. 

Today, with the development of technology, the use of mobile phones, which we see as indispensable, and the transition to smartphones, phone applications are becoming widespread in livestock farming. Waranusast et al. (2017) evaluated the egg size classification on Android mobile devices using image processing and machine learning for determining the physical property of the egg. Egg sizes were classified according to their properties, calculated from the dimensions measured using a support vector machine (SVM) classifier [3].

Lei et al. (2019) proposed a new method that combines a convolutional neural network (CNN) with the heartbeat signal of the hatching eggs for more accurate and effective detection of the hatch rate. They collected the heartbeat signals of the eggs with the PhotoPlethysmoGraphy (PPG) method to detect the change in blood volume in living tissues by photoelectric means. They designed the network E-CNN, used to analyze the order of heartbeat of hatching eggs. They reported that they could determine the fertility rate of hatching eggs with E-CNN and SR-CNN [2].

Similarly, Fadchar and Cruz (2020) established an experimental imaging system to capture an image of five-day-old chicken eggs without damaging the eggshell, for early detection of the fertility status of chicken eggs. They underwent a pre-treatment and color segmentation process to extract the color area parameters of 150 images transferred to the computer [4].

Table 1 lists the success rates of the studies conducted in the literature on egg fertility control.

The studies mentioned in the literature mostly use either classification or segmentation techniques on egg images for feature extraction, and they rarely aim to classify and segment images with the same neural network model. Our study, on the other hand, proposes the use of a single Mask R-CNN-based model with a transfer learning technique to detect, segment and classify fertile and infertile eggs. The proposed study in this article performs the detection, segmentation and classification of fertile and infertile eggs with a single DL model based on the images obtained. In our previous publication [18] presented at the third International Conference on Artificial Intelligence and Applied Mathematics in Engineering (ICAIAME 2021), we attempted to investigate the suitability of Mask RCNN method as a detection and segmentation tool and found that the Mask R-CNN method was quite successful in the problem of egg fertility control.

## 2. Materials and Methods

**Figure 1:**
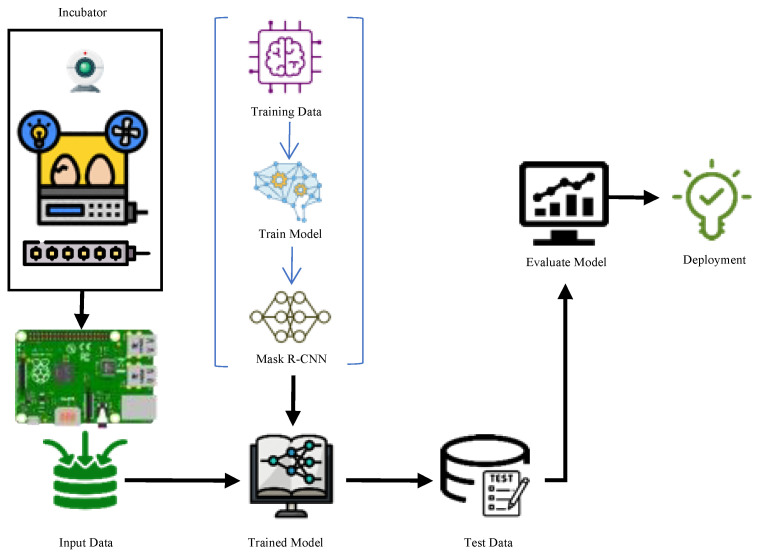
Working phases of the proposed system.

In this study, we aimed to classify chicken eggs according to both segmentation and fertility status with a Mask R-CNN-based approach. For this purpose, first of all, a camera was placed in the incubator, power LEDs were placed under the viols, and a minicomputer was used for the management of the imaging system. The images obtained after the imaging processes were processed on a server computer with high computing power, and the performance of the system was tested. The phases of the proposed system are shown in Figure 1.

**Figure 2:**
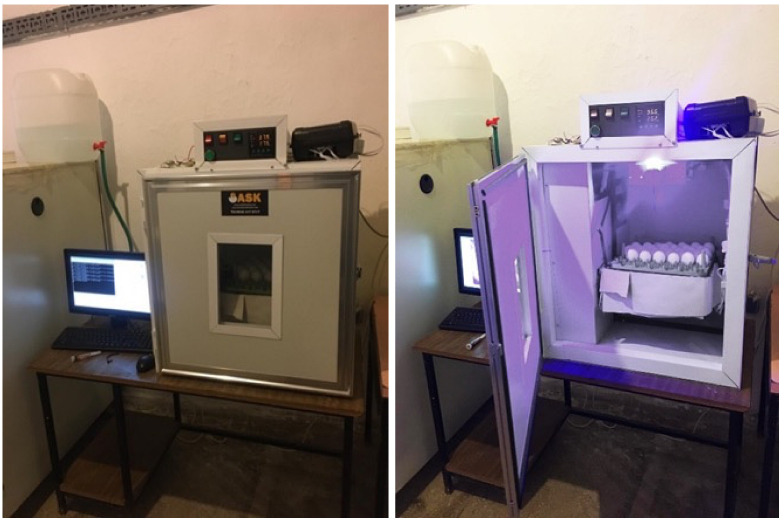
General view of the image acquisition and incubation system.

In the first context, a camera setup was prepared and mounted on the incubator. In this setup, a camera with resolution of 1024 × 768 pixels, an electronic control unit, 30 power LEDs, a step motor for the movement of the camera and a power source are used. Power LEDs were positioned under the trays in which the eggs were placed, lighting was provided from below, and egg images were captured with the camera at the top. Power LEDs are 10 W white light and have an illumination between 6000–6500 K. The camera is positioned at the top of the incubator, and both the camera and the lighting system are controlled by a Raspberry Pi 4 electronic unit. The designed incubator setup is shown in Figure 2.

Obtained egg images were processed on a powerful workstation; they were first segmented and then classified as fertile/infertile by the Mask R-CNN method. The Mask R-CNN method is a deep neural network used to segment objects in computer vision. In the Mask R-CNN technique, which is based on the region-based CNN model, predictions about the regions where an object may be are produced based on the input image. This method predicts the object’s class, refines the bounding box, and creates a pixel-level mask of the object based on the first stage prediction.

The Mask R-CNN method includes a two-step process. These are segmentation and classification. In the segmentation phase, the Region of Interest, which is called RoI, is aligned, and the mask of the object is created according to the spatial plane. This mask is used in the feature mapping of the objects. In the second stage, the class of the object whose boundaries are determined is found.

**Figure 3:**
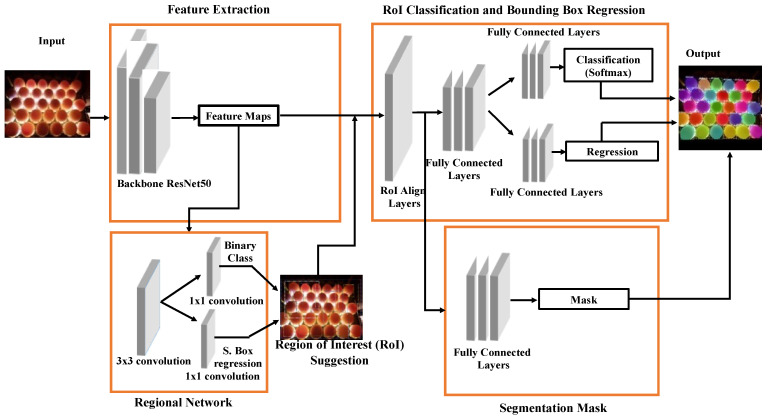
Overview of the Mask R-CNN architecture.

Actually, Mask R-CNN is based on standard R-CNN and is an intuitive extension of Faster R-CNN. It emerged as a solution to the transaction cost problem encountered in R-CNN. In R-CNN, the image is divided into approximately 2000 regions (regions recommended), and CNN (ConvNet) is applied to each region in turn. The size of the regions is determined and placed in the neural network. Since CNN is applied separately to each region in the picture, the training time is very long. Another disadvantage is that it requires a lot of disk space. In order to overcome this, firstly, the Faster R-CNN model is proposed. In this model, the entire image takes a single forward propagation as input in the CNN architecture. In other words, the image is not split according to the region recommendations first. In addition, parts such as ConvNet, RoI pool, and classification layer are combined into one complete architecture. This eliminates the need to store feature maps and saves disk space. Thus, the training process is carried out much faster than R-CNN. Faster R-CNN consists of two stages. The first stage, called the Region Proposal Network (RPN), proposes candidate object bounding boxes. The second stage, essentially Fast R-CNN, extracts features from each candidate box using RoI pool and performs classification and bounding box regression. In the Mask R-CNN model, there is a mask extraction layer in addition to the class label and bounding box offset in the Faster R-CNN architecture. This layer differs from class and box outputs, which require extracting the much finer spatial arrangement of an object [19]. The diagram of the Mask R-CNN method is given in Figure 3.

The higher classification success rate achieved by Hinton’s team in the ImageNet competition in 2012 increased the interest in DNNs. The 26.1% classification success of ImageNet, which is currently used with the name AlexNet, has been reduced to 15.3% by Hinton’s team. The error rates have been further reduced [20] by the architectures developed in the following years (AlexNet-2012 [20], GoogleNet-2014 [21], VGGNet-2014 [22], ResNet-2015 [23], SqueezeNet-2016 [24], NasNet-2017 [25], etc.).

In traditional machine learning methodology, training data and testing data are taken from the same domain, and therefore input feature space and data distribution characteristics are the same, which directly affects the system performance. On the contrary, in some real-world machine learning scenarios where training data are expensive or difficult to obtain, this assumption does not hold. In addition, training processing times of these data are not at acceptable levels for normal users. Therefore, high-performance models (pre-trained networks) that are trained using more easily obtained data from different domains need to be created. This methodology is called transfer learning [26]. In order to use DNN networks with more efficient performance, the ResNet50 pre-trained network model is used as Mask RCNN backbone in this study.

**Figure 4:**
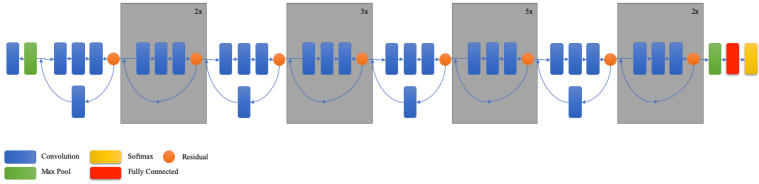
Schematic diagram of ResNet model (compressed view) [31].

ResNet is one of the deepest CNN models in the literature, with 202 trainable layers [27]. It is a DNN network that won first place in the ILSVRC-2015 classification competition. ResNet performs better on CNN models due to its depth [28]. The convolutional layer structure of ResNet has 3*3 filters and this structure is inspired by VGG networks. Two design rules are applied in ResNet: (i) for the same output feature map size, the layers have the same number of filters; and (ii) if the feature map size is halved, the number of filters is doubled in order to preserve the time complexity per layer. The ResNet model has less complexity than the VGG network [22]. The created network was tested using the ImageNet data set as in [22] and [20]. The images were cropped with size 224 × 224, horizontal flip was applied to the images, and then the mean with the per-pixel is subtracted [20]. After each convolution and before activation, batch normalization as in [29] was applied. The weights were determined as in [30] and all residual nets were trained from scratch. The ResNet network consists of different types with layers 18, 34, 50, 101 and 152 (Figure 4). Its main drawbacks are longer training time, training complexity, higher training error, and vanishing gradient of the initial layers in the back-propagation [27].

### 2.1. Evaluate Methods

In the proposed study, object detection, instance segmentation and object classification were conducted. There are many evaluation methods for these processes in the literature. In the evaluation of the tests performed in the study, the intersection over union (IoU) method was used for detection and segmentation, and the maximum accuracy class method was used for classification.

The IoU score is a standard performance measure for the object category segmentation problem. Given a set of images, the IoU measure gives the similarity between the predicted region and the ground-truth region for an object present in the set of images and is defined by the following equation. TP, FP, and FN given in Equation 1 represent true positive, false positive, and false negative, respectively [32].

(1) $$\mathrm{IoU}=\frac{\mathrm{TP}}{\mathrm{FP}+\mathrm{TP}+\mathrm{FN}}$$

When IoU = 0, no overlap exists between the region proposal and the ground truth and then the proposal is accepted as FN. Initially, a threshold IoU is defined to serve as a criterion to decide on a valid region in case more than one bounding box is proposed. When the threshold IoU is 0.5, the region proposals with IoU < 0.5 are predicted as FP (poor prediction) while those with IoU ≧ 0.5 are classified as TP (good prediction). The average of AP values (mAP) is then the metric that gives 1 for IoU values greater than the threshold of 0.5.

An accuracy threshold must be selected when using the IoU as an evaluation metric. For example, in the PASCAL VOC query [33], the commonly reported measure of detection accuracy, i.e., the mean Average Precisions (mAP), is calculated based on a fixed IoU threshold, i.e., 0.5 [34]. The mAP for a set of detection is the mean over classes of the interpolated AP for each class. This class-based AP is specified as the area under the precision/recall (PR) curve for predictions [35].

### 2.2. Implementation

Two different test processes were used in this study. The first test process was carried out between 07 June 2021 and 24 June 2021 (17 days). The second test was carried out between 04 July 2021 and 20 July 2021 (16 Days). In each test procedure, the position angle of the eggs was changed every hour as a requirement of hatching. Unlike the first test, in the second test, the eggs were changed every day to avoid the possibility of the system learning the location of the eggs. In each test process, 24 Denizli chicken eggs were placed in the incubator. These eggs have been selected at random. For this reason, it was determined at the end of the incubation period that there were 5 fertile eggs in the first data set and 18 fertile eggs in the other data set. It was activated with a power LED every 15 min during the incubation period and the images were recorded in JPG format at 1280 × 720 resolution. Python 3.9.6 programming language and Geany compiler were used in Raspberry Pi 4.

The images taken over about 17 days were examined, and the images with different angles, wrong shot, missing light, or noise, etc., were removed. Therefore, 1638 images were obtained for the first data set and 1489 images for the second data set. The data set was used for fertile/infertile egg detection, classification and segmentation in the images. At this stage, the last days of the incubation period were used for the training data and the first days were used for the test data so that the system could offer better accuracy in the separation of the training test data. Accordingly, in the first data set, 1320 images captured between 11 June 2021–24 June 2021 were selected as training data and 317 images captured between 07.06.2021–10.06.2021 were selected as test data. Similarly, in the second data set, 1138 images captured between 09 July 2021–20 July 2021 were selected as training data and 351 images captured between 04 July 2021–08 July 2021 were selected as test data.

**Figure 5:**
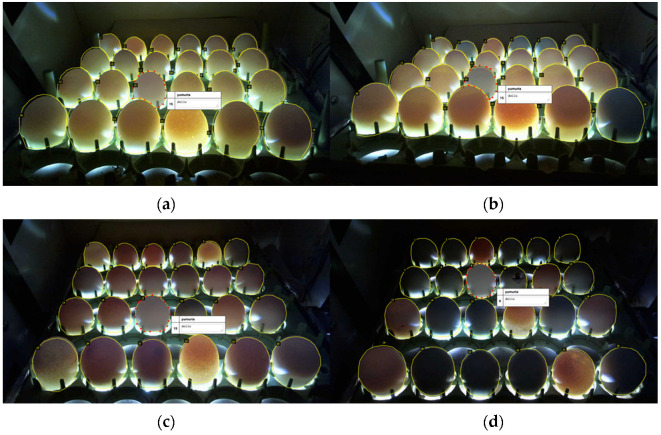
Image labeling processes with the VIA program (Images taken and during the first days (**a**,**b**) and last days (**c**,**d**) of incubation).

The split data sets were labeled with the help of VGG Image Annotator (VIA) [36] during the preprocessing stage. The 24 eggs in each image were individually labeled, so a total of (1638 + 1489) × 24 = 75048 eggs were labeled for the two datasets. In the data sets, since fertile/infertile eggs would be classified, the eggs were labeled according to the fertile/infertile data obtained at the end of the incubation period. A sample screenshot of the VIA program where the labeling process was performed is shown in Figure 5.

The obtained training datasets were trained with Mask R-CNN network structures. For Mask R-CNN application, TensorFlow, and Keras deep learning libraries were used with the help of Python programming. ResNet50 was used as Mask R-CNN backbone. The initial weights of the networks are the COCO model weights. The hyperparameters used in the training phase are given in Table 2.

**Figure 6:**
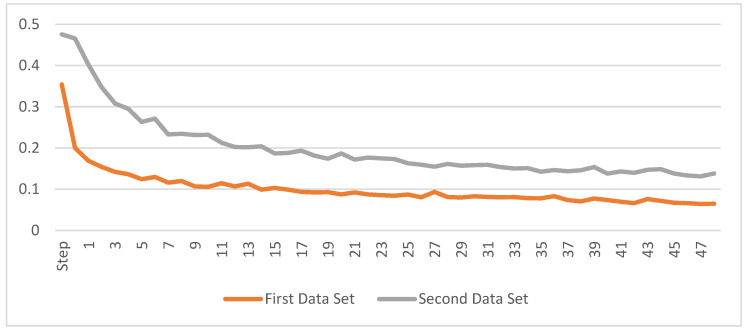
The change of the training loss values.

The computer where the training and testing processes were performed has an AMD Ryzen 3,2200 G 3.5 Hz processor, 8 GB RAM capacity, NVIDIA GeForce GTX 1050 Ti 4 GB Graphics Card (GPU), SSD Plus 240 GB 530 MB–440 MB/s and Windows 11 operating system. Training of the network with ResNet50 backbone lasted about 4.5 h in total for each data set, each epoch was approximately 283 s (~2.8 s/step). The change in the training loss value during the training process for each data set is shown in Figure 6. As can be seen from the graphs, the training loss value for the first data set is below 0.07, and the training loss value for the second data set is below 0.15.

## 3. Results

In this study, in order to detect fertile and infertile eggs, images of egg-placed viols were taken with the help of a camera placed in the incubator. In fertile/infertile detection, the eggs were segmented first and then given as an input to the Mask R-CNN model. The Average Precision (AP) measurement technique was used to control each segmented egg with/without fertility. AP is one of the most important metrics for evaluating object detection and segmentation models in computer vision. If the AP value approaches 1, it means that the segmentation process is successful.

**Figure 7:**
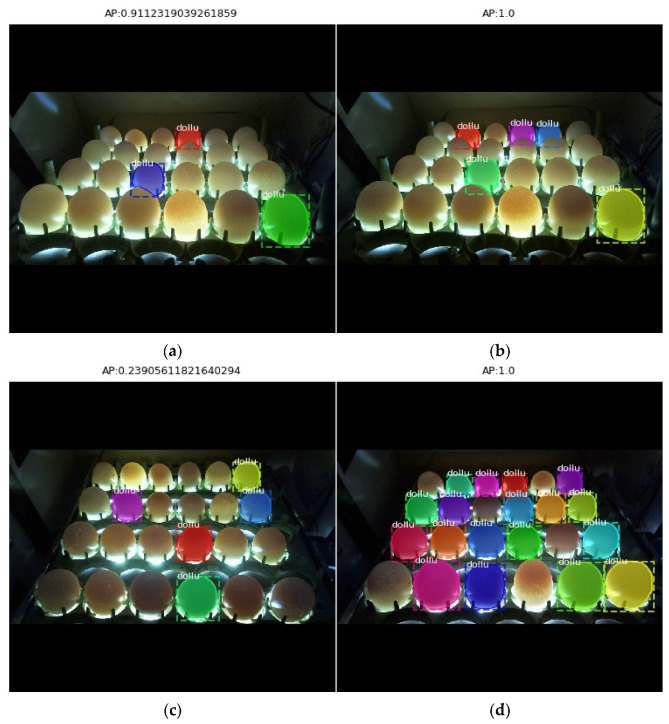
Images resulting from the test process ((**a**,**b**) first dataset—(**c**,**d**) second dataset).

In Figure 7, sample images of the test phase applied to both image databases are provided. Figure 7a,b shows the resulting AP values of the first database containing 317 test images, and Figure 7c,d shows the resulting AP values of the first database containing 351 test images.

**Figure 8:**
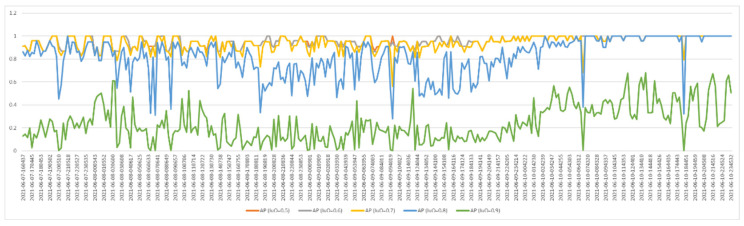
AP values for the first test dataset (IoU: 0.5–0.9).

**Figure 9:**
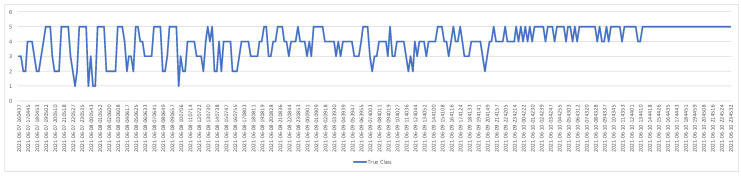
Correct number of fertile eggs for the first test dataset.

As we mentioned in the previous section, the process of taking the egg-placed viol images was performed at a certain time interval (once an hour). Each acquired image was tested and the AP value and correct classification numbers were recorded. Figure 8 and Figure 9 show graphs containing the AP value and correct classification numbers from the tests applied to the first image database, respectively. In this database, five eggs out of 24 are fertile eggs. As can be seen from the graph in Figure 8, the highest AP values were obtained when the IoU value was 0.7. Therefore, fertility control tests were performed by taking IoU = 0.7. When we look at the graph in Figure 9, we see that the number of correct classification varies according to time. When we compare the graphs in Figure 8 and Figure 9, it is seen that the number of correct classification increases and decreases in direct proportion to the success of segmentation. In addition, at the end of the third day, it is understood from the graphs that all five fertile eggs were determined correctly. In other words, for the first data set, fertilized eggs were detected with 100% success at the end of the third day.

**Figure 10:**
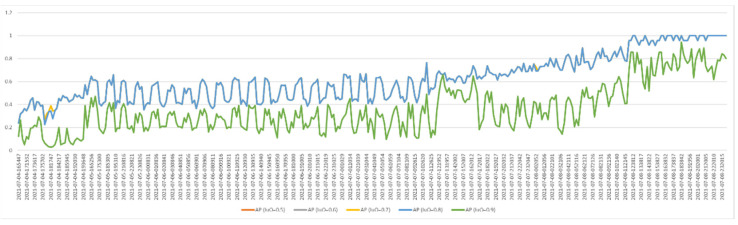
AP values for the second test dataset (IoU: 0.5–0.9).

**Figure 11:**
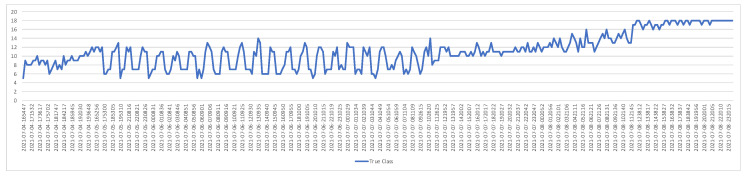
Correct number of fertile eggs for the second test dataset.

Figure 10 and Figure 11 show graphs containing the AP value and correct classification numbers from the tests applied to the second image database, respectively. In this database, 18 eggs out of 24 are fertile eggs. When we analyze the AP values, it is seen that the highest performance is obtained when the IoU value is 0.7 and 0.8. The graph in Figure 11 includes the number of fertile eggs correctly determined as a result of the tests performed by taking IoU = 0.7. In these two graphs, as in the previous one, it is seen that the segmentation and correct detection performances vary in parallel with each other. Furthermore, similar to the previous study, all fertile eggs (18 eggs) were detected correctly at the end of the third day. As a result of the test with the second data set, all fertilized eggs were detected with 100% success at the end of the third day.

## 4. Conclusions

Our proposed system for the fertile/infertile control of chicken eggs includes image processing and deep learning techniques. First of all, viol images of 24 eggs were taken with the help of a camera placed in the incubator, and then the segmentation process was implemented. Two image databases, containing 5 and 18 fertile eggs, were created and segmentation and fertilized egg detection processes were carried out, respectively, with a Mask R-CNN approach.

When the literature on egg fertility control is examined, the studies mostly use either classification or segmentation techniques on egg images for feature extraction, and they rarely aim to classify and segment images with the same neural network model. Our study, on the other hand, proposes the use of a single model to detect, segment and classify fertile and infertile eggs. When the results obtained in our study are examined, for the 0.7 IoU threshold value, it can be seen that the fertility control can be conducted correctly on the third day. Likewise, it was observed that the AP value was 1 on the third day. Considering the methods used and the ease of the designed system, it can be said that a very successful system has been designed according to the studies in the literature.

IoU and AP measurement metrics, which are accepted in the literature, were used to evaluate the segmentation performance. It can be seen from our study that AP measurement values are higher when IoU = 0.7 is selected. According to the test results of the segmentation and fertile egg detection process we applied to both image databases, all fertile eggs can be detected correctly at the end of the third day. However, when the data we received from the tests are analyzed, the segmentation performance has a great effect on the correct detection of the fertile egg because the AP values obtained in the segmentation and the number of correct detection of fertile eggs were parallel to each other.

So, in fact, improving the segmentation performance is much more important than the detection of fertile eggs. For this, in other words, in order to increase the segmentation performance, it is necessary to carry out an experimental study to improve the camera and lighting setup prepared for taking the images. In the continuation of this study, we aim to develop the image acquisition mechanism.

The method proposed in this study can be used for the fertility control of all poultry eggs. A commercial product has not been developed yet, but we continue to work on the development of a commercial product using the method we propose.

## Figures and Tables

**Table 1:** Egg fertility control literature review.

Author(s), Date	Method(s)	Success Rates	Achieved Day
K. Das and M. Evans, 1992 [6,7]	Histogram, Characterization and Neural Network Classifier	93% 88–90%	At the end of the 3rd day At the end of the 3rd day
F. Bamelis, K. Tona, J. De Baerdemaeker, and E. Decuypere, 2002 [8]	Spectrophotometric Method	-	4.5th day
Y. Usui, K. Nakano, and Y. Motonaga, 2003 [9]	Halogen Light Source and NIR Detection System	83–96.8%	-
K. C. Lawrence, D. P. Smith, W. R. Windham, G. W. Heitschmidt, and B. Park, 2006 [10,11]	Hyperspectral Imaging Technique	91%	At the end of the 3rd day
D. Smith, K. Lawrence, and G. Heitschmidt, 2006 [12]	Mahalanobis Distance (MD) Classification and Partial Least Squares Regression (PLSR)	96% (MD), 100% (PSLR) 92% (MD), 100% (PSLR) 100% (MD), 100% (PSLR)	At the end of the 0th day At the end of the 1st day At the end of the 2nd day
Chern-Sheng Lin, Po Ting Yeh, Der-Chin Chen, Yih-Chih Chiou, Chi-Hung Lee, 2013 [13]	Thermal Images and Fuzzy System	96%	-
L. Liu & M. O. Ngadi, 2013 [14]	Near Infrared Hyperspectral Images, PCA, K-Means	100% 78.8% 74.1% 81.8%	At the end of the 0th day At the end of the 1st day At the end of the 2nd day At the end of the 4th day
Waranusast ve ark., 2017 [3]	Image processing and machine learning (SVM)	80.4%	-
Boga et al., 2019 [15]	Image processing with thresholding	73.34% (1st dataset) 100% (1st dataset) 93.34% (2nd dataset) 93.34% (2nd dataset) 93.34% (3rd dataset) 100%(3rd dataset)	At the end of the 3rd day At the end of the 4th day At the end of the 3rd day At the end of the 4th day At the end of the 3rd day At the end of the 4th day
Huang et al. [16]	Deep Convolutional Neural Network	98.4%	five- to seven-day embryos
Geng et al. [17]	Deep convolutional neural networks	98.3% 99.1%	At the end of the 5th day At the end of the 9th day
Lei et al., 2019 [2]	PhotoPlethysmoGraphy (PPG), convolutional neural network (CNN)	99.50%	-
Glenn ve ark., 2019 [1]	Fuzy Logic and k-nearest neighbors (k-NN)	N/A	-
Fadchar and Cruz, 2020 [4]	Color segmentation and artificial neural network (ANN)	97%	-

**Table 2:** Hyperparameters used in model training.

Hyperparameters	Values
Optimizer	ADAM
Epoch	50
Step in each epoch	100
Batch size	1
Learning rate	0.001
Coefficient of determination	0.9

## Data Availability

Not applicable.
